# Supplementary material for: Promoting Fc-Fc interactions between anti-capsular antibodies provides strong immune protection against Streptococcus pneumoniae
Source: eLife. 2023 Mar 22;12:e80669. doi: 10.7554/eLife.80669 (PMC10032657; doi:10.7554/eLife.80669)
Supplement: Supplementary file 1. — Variable and constant heavy and light chain protein sequences used for antibody production. The residues E345 and E430 are highlighted in light gray and dark gray, respectively. The adapted amino acids for this study are highlighted in red. [file elife-80669-supp1.docx]

**SUPPLEMENTARY INFORMATION - Promoting Fc-Fc interactions between anti-capsular antibodies provides strong complement-dependent immune protection against *Streptococcus pneumoniae***

Leire Aguinagalde^1^, Maurits A. den Boer^2,3^, Suzanne M. Castenmiller^1^, Seline A. Zwarthoff^1^, Carla J.C Gosselaar-de Haas^1^, Piet C. Aerts^1^, Frank J. Beurskens^4^, Janine Schuurman^4^, Albert J.R. Heck^2,3^, Kok P.M. van Kessel^1^ and Suzan H.M. Rooijakkers^1*^

*^1^Medical Microbiology, University Medical Center Utrecht, Utrecht University, The Netherlands*

*^2^Biomolecular Mass Spectrometry and Proteomics, Bijvoet Center for Biomolecular Research and Utrecht Institute for Pharmaceutical Sciences, Utrecht University, Padualaan 8, 3584 CH Utrecht, The Netherlands*

*^3^Netherlands Proteomics Center, Padualaan 8, 3584 CH Utrecht, The Netherlands*

*^4^Genmab, Utrecht, The Netherlands*

**Supplementary file 1: Protein sequences used for antibody production.**

|  | **Sequence** | |
| --- | --- | --- |
| **Variable heavy chain** | | |
| CPS6-IgG | EVQLVESGGGLVTFGGSLTLSCAASGFTFSRAWLTWVRQAPGGGLEWVGRILRMADGGATDYAATVKGRFTISRDDSKNTVYLHMNNLKTEDTAVYYCANENFWRLDNWGQGTLVTVSS | |
| CPS8-IgG(28H11) | EVKLLESGGGLVQPGGSLKLSCAASGFDFSRYWMSWVRQAPGKGLEWIGEINPDSSTINYTPSLKDKFIISRDNAKNTLYLQMSKVRSEDTALYYCARPWFRYFDVWGAGTTVTVSS | |
| CPS3-IgG(5F6) | QVQLQQPGAELVRPGASVKLSCKASGYTFTSYWINWMKQRPGQG LEWIGVIDPSDSETHYNQMFKDKATLTVDKSSSTAYMQLSSLTSEDSAVYYCARRGDDGYTPAWFAYWGQGTLVTVSA | |
| **Variable light chain** | | |
| CPS6-IgG | QSALTQPASVSGSPGQSITISCTGTSSDVGTYNHVSWYQQCPGKGPKLLIYDVTNRPSGVSNRFSGSKSDNTASLTISGLQAEDEADYYCYSNTASGAPYVFGSGTKVTVLSQPKANPTVTLFPPSS | |
| CPS8-IgG(28H11) | DIVMTQSPSSLAMSVGQKVTMSCKSSQSLLNSSNQKNYLAWYQQKPGQSPKLLVYFASTRESGVPDRFIGSGSGTDFTLTISSVQAEDLADYFCQQHYSTPYTFGGGTKLEIK | |
| CPS3-IgG(5F6) | DVVVTQTPLSLPVSLGDQASISCRSSQSLLHSNGNTYLHWYLQK PGQSPKLLIYKVSNRFSGVPDRFSGSGSGTDFTLKISRVEAEDLGVYFCSQSTHVPTF GGGTKLEIK | |
| **Constant heavy chain** | | |
| IgG1 | ASTKGPSVFPLAPSSKSTSGGTAALGCLVKDYFPEPVTVSWNSGALTSGVHTFPAVLQSSGLYSLSSVVTVPSSSLGTQTYICNVNHKPSNTKVDKKVEPKSCDKTHTCPPCPAPELLGGPSVFLFPPKPKDTLMISRTPEVTCVVVDVSHEDPEVKFNWYVDGVEVHNAKTKPREEQYNSTYRVVSVLTVLHQDWLNGKEYKCKVSNKALPAPIEKTISKAKGQPREPQVYTLPPSREEMTKNQVSLTCLVKGFYPSDIAVEWESNGQPENNYKTTPPVLDSDGSFFLYSKLTVDKSRWQQGNVFSCSVMHEALHNHYTQKSLSLSPGK | |
| IgG2 | ASTKGPSVFPLAPCSRSTSESTAALGCLVKDYFPEPVTVSWNSGALTSGVHTFPAVLQSSGLYSLSSVVTVPSSNFGTQTYTCNVDHKPSNTKVDKTVERKCCVECPPCPAPPVAGPSVFLFPPKPKDTLMISRTPEVTCVVVDVSHEDPEVQFNWYVDGVEVHNAKTKPREEQFNSTFRVVSVLTVVHQDWLNGKEYKCKVSNKGLPAPIEKTISKTKGQPREPQVYTLPPSREEMTKNQVSLTCLVKGFYPSDIAVEWESNGQPENNYKTTPPMLDSDGSFFLYSKLTVDKSRWQQGNVFSCSVMHEALHNHYTQKSLSLSPGK | |
| IgG3 | ASTKGPSVFPLAPCSRSTSGGTAALGCLVKDYFPEPVTVSWNSGALTSGVHTFPAVLQSSGLYSLSSVVTVPSSSLGTQTYTCNVNHKPSNTKVDKRVELKTPLGDTTHTCPRCPEPKSCDTPPPCPRCPEPKSCDTPPPCPRCPEPKSCDTPPPCPRCPAPELLGGPSVFLFPPKPKDTLMISRTPEVTCVVVDVSHEDPEVQFKWYVDGVEVHNAKTKPREEQYNSTFRVVSVLTVLHQDWLNGKEYKCKVSNKALPAPIEKTISKTKGQPREPQVYTLPPSREEMTKNQVSLTCLVKGFYPSDIAVEWESSGQPENNYNTTPPMLDSDGSFFLYSKLTVDKSRWQQGNIFSCSVMHEALHNRFTQKSLSLSPGK | |
| **J-chain** | | |
| IgM | QEDERIVLVDNKCKCARITSRIIRSSEDPNEDIVERNIRIIVPLNNRENISDPTSPLRTRFVYHLSDLCKKCDPTEVELDNQIVTATQSNICDEDSATETCYTYDRNKCYTAVVPLVYGGETKMVETALTPDACYPD | |
| **Constant light chain (*kappa* class)** | | |
| IgG1,2,3, | RTVAAPSVFIFPPSDEQLKSGTASVVCLLNNFYPREAKVQWKVDNALQSGNSQESVTEQDSKDSTYSLSSTLTLSKADYEKHKVYACEVTHQGLSSPVTKSFNRGEC | |
| **Constant light chain (*lambda* class)** | | |
| IgG1 (CPS6) | | EELQANKATLVCLISDFYPGAVTVAWKADGSPVKAGVETTKPSKQSNNKYAASSYLSLTPEQWKSHRSYSCQVTHEGSTVEKTVAPTECS |
